# The Arp2/3 Inhibitory Protein Arpin Is Required for Intestinal Epithelial Barrier Integrity

**DOI:** 10.3389/fcell.2021.625719

**Published:** 2021-05-03

**Authors:** Sandra Chánez-Paredes, Armando Montoya-García, Karla F. Castro-Ochoa, Julio García-Cordero, Leticia Cedillo-Barrón, Mineko Shibayama, Porfirio Nava, Sven Flemming, Nicolas Schlegel, Alexis M. Gautreau, Hilda Vargas-Robles, Ricardo Mondragón-Flores, Michael Schnoor

**Affiliations:** ^1^Department of Molecular Biomedicine, CINVESTAV-IPN, Mexico City, Mexico; ^2^Department of Infectomics and Molecular Pathogenesis, CINVESTAV-IPN, Mexico City, Mexico; ^3^Department of Physiology, Biophysics and Neurosciences, CINVESTAV-IPN, Mexico City, Mexico; ^4^Department of Surgery I, University Hospital Würzburg, Würzburg, Germany; ^5^CNRS UMR 7654, Institut Polytechnique de Paris, Palaiseau, France; ^6^Department of Biochemistry, CINVESTAV-IPN, Mexico City, Mexico

**Keywords:** actin cytoskeleton, colitis, intestinal barrier, ZO-1, ulcerative colitis, inflammatory bowel diseases, tight junction, mesalazine (5-aminosalicylic acid)

## Abstract

The intestinal epithelial barrier (IEB) depends on stable interepithelial protein complexes such as tight junctions (TJ), adherens junctions (AJ), and the actin cytoskeleton. During inflammation, the IEB is compromised due to TJ protein internalization and actin remodeling. An important actin regulator is the actin-related protein 2/3 (Arp2/3) complex, which induces actin branching. Activation of Arp2/3 by nucleation-promoting factors is required for the formation of epithelial monolayers, but little is known about the relevance of Arp2/3 inhibition and endogenous Arp2/3 inhibitory proteins for IEB regulation. We found that the recently identified Arp2/3 inhibitory protein arpin was strongly expressed in intestinal epithelial cells. Arpin expression decreased in response to tumor necrosis factor (TNF)α and interferon (IFN)γ treatment, whereas the expression of gadkin and protein interacting with protein C-kinase α-subunit 1 (PICK1), other Arp2/3 inhibitors, remained unchanged. Of note, arpin coprecipitated with the TJ proteins occludin and claudin-1 and the AJ protein E-cadherin. Arpin depletion altered the architecture of both AJ and TJ, increased actin filament content and actomyosin contractility, and significantly increased epithelial permeability, demonstrating that arpin is indeed required for maintaining IEB integrity. During experimental colitis in mice, arpin expression was also decreased. Analyzing colon tissues from ulcerative colitis patients by Western blot, we found different arpin levels with overall no significant changes. However, in acutely inflamed areas, arpin was significantly reduced compared to non-inflamed areas. Importantly, patients receiving mesalazine had significantly higher arpin levels than untreated patients. As arpin depletion (theoretically meaning more active Arp2/3) increased permeability, we wanted to know whether Arp2/3 inhibition would show the opposite. Indeed, the specific Arp2/3 inhibitor CK666 ameliorated TNFα/IFNγ-induced permeability in established Caco-2 monolayers by preventing TJ disruption. CK666 treatment also attenuated colitis development, colon tissue damage, TJ disruption, and permeability in dextran sulphate sodium (DSS)-treated mice. Our results demonstrate that loss of arpin triggers IEB dysfunction during inflammation and that low arpin levels can be considered a novel hallmark of acute inflammation.

## Significance to the Field

Intestinal epithelial barrier dysfunction is a hallmark of inflammatory bowel diseases, but the underlying mechanisms remain poorly understood. Interepithelial junctions that are connected to the actin cytoskeleton stabilize cell contacts. While the role of junctional transmembrane and scaffolding proteins for barrier integrity has been extensively studied, the role of actin-binding proteins in this context is still poorly understood. Thus, it is critical to improve our understanding of how actin-binding proteins regulate both TJ and cytoskeleton architecture during inflammatory disorders. Here, we provide *in vivo*, *in vitro*, and *in situ* evidence that the actin-branching nucleator Arp2/3 and its regulatory protein arpin are critical for maintaining proper barrier functions in the colon under basal and inflammatory conditions. Our data suggest that loss of arpin is a new hallmark of acute inflammation in ulcerative colitis and that the Arp2/3 complex may serve as a therapeutic target to treat inflammatory bowel diseases.

## Introduction

The intestinal epithelium is a single layer of cells lining the gut lumen that not only provides a physical barrier against luminal bacteria and antigens but also regulates absorption and diffusion of water, nutrients, and ions. Epithelial cells are joined together by tight junctions (TJ), adherens junctions (AJ), and desmosomes (Turner, 2009). Under inflammatory conditions as occurring for example in ulcerative colitis (UC), the intestinal epithelium gets compromised by junction protein internalization and actin cytoskeletal remodeling (Utech et al., 2010; Luissint et al., 2016; Lechuga and Ivanov, 2017). An important actin regulator responsible for the formation of branched actin filaments is the highly conserved heptameric actin-related protein 2/3 (Arp2/3) complex (Rotty et al., 2013). The subunits Arp2, Arp3, ArpC1, ArpC2, ArpC3, ArpC4, and ArpC5 assemble the Arp2/3 complex. Of note, the presence of one subunit means the presence of the whole functional complex, as subunits are unstable in their uncomplexed form (Mullins et al., 1997; Molinie and Gautreau, 2018). The Arp2/3 complex has a low basal activity and requires interaction with nucleation-promoting factors (NPFs) to become fully activated. A strict regulation of Arp2/3 activity is guaranteed by proteins inhibiting Arp2/3 (PIAs) that antagonize NPF in certain cellular substructures (Molinie and Gautreau, 2018). For example, Wiskott–Aldrich syndrome protein (WASP)-family verprolin-homologous protein (WAVE) activates Arp2/3 at lamellipodia, where it is antagonized by arpin (Dang et al., 2013). At clathrin-coated pits, Arp2/3 activity is triggered by neural Wiskott–Aldrich syndrome protein (N-WASP) and negatively modulated by protein interacting with protein C-kinase α-subunit 1 (PICK1) (Rocca et al., 2008). At endosomes, Arp2/3 is activated by Wiskott–Aldrich syndrome protein and SCAR homolog (WASH) and inhibited by gadkin (γ-1 and kinesin interactor) (Maritzen et al., 2012). The importance of the Arp2/3 complex and its activator WAVE in epithelial barrier formation has been well studied (Verma et al., 2004, 2012; Zhou et al., 2013, 2015). Moreover, in endothelial cells, Arp2/3 and WAVE mediate junction stability through temporal formation of junction-associated intermittent lamellipodia (JAIL) (Abu Taha et al., 2014). During inflammation, Arp2/3-dependent lamellipodia participate in healing endothelial micro-wounds induced by transmigrating leukocytes (Martinelli et al., 2013). In the epithelium, lamellipodia drive the restoration of barrier integrity *in vitro* and *in vivo* (Begnaud et al., 2016). Arp2/3 is also involved in epithelial junction regulation in the *Drosophila* notum, where it contributes to junction protein internalization (Georgiou et al., 2008). Thus, fine-tuning Arp2/3 activity at junctions seems to be critical for proper epithelial barrier functions, but it is currently unknown how this is achieved. A possibility is that PIAs control Arp2/3 activity at epithelial junctions in competition with NPF (Chanez-Paredes et al., 2019). The most recently identified PIA is arpin that competes with the WAVE complex in lamellipodia to regulate random cell migration (Dang et al., 2013), but not chemotaxis (Dang et al., 2017). While the importance of arpin has been shown in different cancer types (Liu et al., 2016; Lomakina et al., 2016; Li T. et al., 2017; Li Y. et al., 2017; Zhang et al., 2019), nothing is known about arpin functions in intestinal epithelium and in epithelial barrier regulation during inflammatory disorders including UC.

We hypothesized that Arp2/3 is locally inhibited by arpin to regulate barrier integrity by controlling actin cytoskeleton and TJ architecture. To test this hypothesis, we employed two different strategies: (1) depleting the endogenous PIA arpin and (2) using the well-established specific pharmacological Arp2/3 inhibitor CK666.

## Materials and Methods

### Cell Culture

The colorectal adenocarcinoma epithelial Caco-2 (clone C2BBE1) cell line was obtained from ATCC (Manassas, VA, United States) and cultured according to their instructions. Confluent Caco-2 monolayers were treated with 50 ng/ml tumor necrosis factor (TNF)α (Peprotech, Mexico) and 10 ng/ml interferon (IFN)γ (Peprotech, Mexico) and incubated for 48 h in a humidified atmosphere with 5% CO_2_ at 37°C to mimic inflammation.

### End-Point PCR and Quantitative RT-PCR

Total RNA was isolated using TRIzol reagent, quantified using a NanoDrop ND-1000 spectrophotometer, and treated with RNase-free DNase I. RNA was reverse-transcribed using oligo-dT primers and SuperScript II reverse transcriptase according to the manufacturer’s instructions. *End-point PCR* was performed using Platinum^®^, PCR SuperMix, 0.15 μM forward primers, 0.15 μM reverse primers (Supplementary Table 1), and 100 ng cDNA. PCR conditions were as follows: 95°C for 3 min, followed by 35 cycles of 95°C for 30 s, 55°C for 30 s, 72°C for 30 s, and a final extension at 72°C for 10 min and 4°C ∞. The PCR products were separated by electrophoresis on 2% agarose gels. All reagents were from Thermo Fisher Scientific (Waltham, MA, United States).

qRT-PCR was carried out in a total volume of 10 μl, containing 5.0 μl Power SYBR Green PCR Master Mix 2 × (Applied Biosystems, Foster City, CA, United States), 0.15 μM forward primer, 0.15 μM reverse primer, and 100 ng cDNA using a StepOne^TM^ Real-Time PCR System (Applied Biosystems). Conditions were as follows: activation at 95°C for 10 min, 40 cycles including denaturation phase at 95°C for 15 s, and data acquisition during the annealing/extension step at 60°C for 60 s. Following the last cycle, the melting curve was generated by heating from 60°C to 95°C in increments of 0.6°C/s. Relative expression was quantified using the 2^–ΔΔ*CT*^ method (Rao et al., 2013).

Human *7SL* (Galiveti et al., 2010) and mouse *Actb* were used as housekeeping genes.

### Western Blot

Radioimmunoprecipitation assay (RIPA) buffer cell and tissue lysates were separated by sodium dodecyl sulfate-polyacrylamide gel electrophoresis (SDS-PAGE), transferred to 0.45-μm-pore nitrocellulose membranes (Bio-Rad, Hercules, CA, United States), blocked with Tris-buffered saline (TBS) containing 0.05% Tween (TBS-T) and 5% skim milk, or 5% bovine serum albumin (BSA) for myosin-II light chain (MLC) and phosphorylated-MLC (p-MLC), for 1 h at room temperature (RT), and incubated overnight in primary antibodies at 4°C with gentle agitation. Membranes were washed with TBS-T three times for 10 min each and incubated with species-specific secondary antibodies conjugated to horseradish peroxidase for 1 h at RT with gentle agitation. After another three washes for 10 min each, bands were visualized using SuperSignal West Pico substrate (Thermo Fisher Scientific) and a ChemiDoc device (Bio-Rad, Hercules, CA, United States). Pixel intensity was quantified using ImageJ software (NIH, Bethesda, MD, United States). Primary and secondary antibodies are listed in Supplementary Table 2. Full blots including the molecular weight markers are shown in Supplementary Figure 1.

### Co-immunoprecipitation

10 μl of Protein G-sepharose beads (Sigma-Aldrich) were equilibrated in 500 μl of lysis buffer (50 mM Tris-HCl pH 7.4, 150 mM NaCl, 1 mM EDTA, 1% NP-40, and 2 × cOmplete^TM^ protease cocktail and 2 × PhosSTOP^TM^) and shaken gently for 4 h at 4°C. Cell extracts (1 mg protein) were pre-cleared with equilibrated beads for 2 h at 4°C. Then, beads were pelleted at 3,500 rpm for 5 min at 4°C, and pre-cleared extracts were incubated overnight at 4°C under gentle rotation with primary antibodies. Unrelated serum was used as a negative control. The next day, 10 μl of equilibrated G-agarose beads blocked with lysis buffer containing 4% BSA were added to the antibody–antigen complex and incubated overnight at 4°C with gentle rotation. The antibody–antigen–beads conjugates were separated by centrifugation at 3,500 rpm for 5 min at 4°C, washed three times with 1 × phosphate-buffered saline (PBS) and eluted with Laemmli buffer. Samples were denatured by boiling for 5 min at 95°C and analyzed by Western blot.

### Immunofluorescence Microscopy

Cells on glass coverslips were fixed either in 4% paraformaldehyde (PFA) for 10 min at RT and permeabilized with 0.2% Triton X-100 in PBS for 5 min at RT or in absolute ethanol for 30 min at −20°C. Coverslips were then blocked for 20 min in PBS containing 3% BSA. Primary antibodies were incubated overnight at 4°C. Then, coverslips were washed and incubated with species-specific fluorescently labeled secondary antibodies for 1 h at RT. Coverslips were mounted in Prolong medium containing 4’,6-diamidino-2-phenylindole (DAPI; Thermo Fisher Scientific) and analyzed using a confocal laser microscope (Leica TCS, SPE).

### Transmission Electron Microscopy

Cells were processed as previously reported (Muniz-Hernandez et al., 2011). Briefly, cells were grown in Petri dishes to a confluency of about 90% and fixed with 2.5% glutaraldehyde for 1 h. After thorough washings with PBS, cells were carefully scraped off, rinsed with PBS, and postfixed for 1 h in 1% OsO_4_ at 4°C. Subsequently, cells were dehydrated with increasing concentrations of ethanol, embedded in Spurr’s resin (Electron Microscopy Sciences, Washington, DC, United States), and polymerized at 60°C for 48 h. Ultrathin sections were cut using an Ultracut E ultramicrotome (Reichert-Jung, Wien, Austria), stained with uranyl acetate and lead citrate, and analyzed using a JEOL 1400 electron microscope (JEOL LTD, Japan). Quantification of microvilli and vesicle numbers and TJ length was done in at least six cells of each tested condition from two independent experiments. To this end, frames were drawn at intercellular junctions (5 × 2 μm) and along the entire apical membrane of single cells in order to delimit zones for counting of junction-related vesicles and microvilli, respectively.

### Transepithelial Electrical Resistance Measurement

Here, 6 × 10^5^ Caco-2 cells were seeded on 0.4-μm pore size Transwell filters (Corning-Costar, Acton, MA, United States). Transepithelial electrical resistance (TER) was measured using a Milicell-Electrical Resistance System (Milicell-ERS) MERS00001. TER is presented as Ω × cm^2^ or as relative TER normalized to control cells. In the experiments using CK666 during the formation of monolayers, culture medium containing CK666 or dimethylsulfoxide (DMSO) alone was exchanged every 2 days.

### Paracellular Flux Assay

At the end of each TER assay, monolayers were rinsed three times with pre-warmed Hanks’ balanced salt solution (HBSS) supplemented with 4-(2-hydroxyethyl)-1-piperazineethanesulfonic acid (HEPES), glucose, and Ca^2+^/Mg^2+^. Then, 100 μg of fluorescein isothiocyanate (FITC)-dextran (4 kDa, Sigma-Aldrich, St. Louis, MO, United States) were added to the apical side and incubated for 2 h at 37°C. Medium from the bottom chamber was collected, and the amount of diffused dextran was measured using a fluorometer. Emission values were normalized to control cells (set to 100%).

### Generation of Arpin-Depleted Cells

A stable arpin-depleted Caco-2 cell line was generated by lentiviral transduction using the trans-lentiviral packaging kit (Thermo Fisher Scientific) and the pLKO.1 plasmid (Addgene, Cambridge, MA, United States) according to the manufacturer’s instructions. The following shRNA sequences were used: shRNA scrambled: 5′-CGGAGAAGTGGAGAAGCATAC-3′ and shRNA arpin: 5′-GGAGAACTGATCGATGTATCT-3′ (Dang et al., 2013). shRNA insertion after cloning was verified by sequencing.

### Actin Density Quantification

Actin density was quantified using ImageJ (NIH, Bethesda, Maryland). Briefly, maximum projections of confocal stacks were converted to 8-bit format. Then, five cells from each of four independent experiments were delineated with the “free selection tool.” Area, mean gray value, and integrated density were measured for each cell and for three random background fields. Corrected density was calculated using the following formula: *Corrected density* = *integrated density – (Area ^∗^ mean gray of background)*. Resulting data were normalized to the average of shCtrl cells.

### Animal Studies

All experiments have been approved by the Institutional Animal Care and Use Committee of CINVESTAV-IPN. Animals were handled according to Mexico’s official norm NOM-062-ZOO-1999 established for the production, care, and use of laboratory animals in agreement with international standards. Adult male C57BL/6 mice (20–25 g) were used in colitis experiments. All animals were given *ad libitum* access to standard pellet diet and water over the entire experimental period of 7 days. The colitis group received 2.5% dextran sulphate sodium (DSS; molecular mass 40 kDa; Carbosynth, CA, United States) in drinking water. To assess the severity of colitis, the disease activity index (DAI) was scored daily from 0 to 4 based on body weight loss, diarrhea, and intestinal bleeding (Mennigen et al., 2009; Park et al., 2013). For CK666 administration, groups of mice were intraperitoneally (i.p.) injected with 5 mg/kg CK666 (Park et al., 2013; Li et al., 2018) at days 3, 4, 5, and 6 of colitis induction or 10% DMSO in PBS as control. The control groups received normal drinking water and the same daily dose of DMSO or CK666. After 7 days, animals were euthanized, and the colon and ileum were removed, measured, and then used in further experiments.

### Histology

Cross sections of colon Swiss rolls (Bialkowska et al., 2016) embedded in paraffin and mounted on glass slides were stained with hematoxylin and eosin according to standard protocols. A general histology score was determined by a pathologist in a blinded fashion, taking into account the degree of inflammation, extent of inflammation, and crypt damage in relation to the percentage of epithelium involved (Mennigen et al., 2009). Also, a more detailed score including loss of goblet cells, cryptitis, hyperemia, lamina propria inflammation, epithelial erosion, mucosal edema, crypt dropout, and architectural distortion was determined as published previously (Shukla et al., 2018).

### *In vivo* Intestinal Epithelial Permeability

Animals were anesthetized by i.p. injection of ketamine/xylazine (100 mg/kg and 13 mg/kg of body weight, respectively) in 0.9% saline solution. After laparotomy, colons were exposed, and a G22-polyethylene tube was inserted into the colon *ascendens*. The colon was flushed with PBS followed by instillation of 1.5% Evans blue solution. The dye was incubated for 15 min, then mice were euthanized, and colons were removed, rinsed with abundant PBS, and washed with 1 ml of 6 mM N-acetylcysteine in PBS to eliminate excess dye. Colon weight was recorded, the dye was extracted overnight at RT in 2 ml of N, N dimethylformamide and measured spectrophotometrically at 610 nm (Vargas Robles et al., 2017).

### Human Tissue Samples

Human tissue samples were obtained from UC patients who underwent surgical resection. The study was approved by the ethics committee of the University of Würzburg (protocol numbers 113/13, 46/11, 42/16) (Meir et al., 2019), and written informed consent was obtained from all patients. Control tissue samples were from surgical colon resections from patients with colon carcinoma, as these resections routinely involve removal of a large part of healthy, uninflamed colon surrounding the tumor. For our experiments, we only used resected tissue far away from the tumor that has no spatial relation with the tumor and can thus be considered healthy (Meir et al., 2019). Control tissue was used only from patients who did not suffer from UC or Crohn’s disease. Patient characteristics are summarized in Table 1. For Western blot, tissue samples were taken immediately after resection and lysed in SDS lysis buffer containing 25 mM HEPES, 2 mM ethylenediaminetetraacetic acid (EDTA), 25 mM sodium fluoride (NaF), and 1% SDS. Another part of the tissue was fixed in 4% PFA, embedded in paraffin, sectioned, and stained as described above.

**TABLE 1:** Clinical data from control and ulcerative colitis (UC) patients.

Sample	Sex	Age	Medication	Histology	Location	Arpin	ArpC5
Ctrl 1	F	60	None	Colon ascendens carcinoma	Colon ascendens	0.9613	1.0094
Ctrl 2	M	82	None	Colon ascendens carcinoma	Colon ascendens	1.3864	0.9304
Ctrl 3	M	49	None	High-grade IEN cecum	Colon ascendens	1.0732	0.8729
Ctrl 4	F	72	None	Colon ascendens carcinoma	Colon ascendens	1.0737	1.0338
Ctrl 5	M	76	None	Colon ascendens carcinoma	Colon ascendens	1.0084	1.1396
Ctrl 6	M	72	None	Rectal cancer	Colon descendens	1.1629	1.2536
Ctrl 7	F	61	None	Carcinoma of rectosigmoidal transition	Colon descendens	1.1048	1.0917
Ctrl 8	M	91	None	Colon ascendens carcinoma	Colon ascendens	1.2847	0.8669
UC 1	M	62	None	Colitis-associated carcinoma, active UC	Colon ascendens	0.6556	1.0374
UC 2	M	54	Budenoside, mesalazine	Active UC, neuroendocrine tumor	Rectum	1.0147	1.0622
UC 3	M	53	Adalimumab, prednisolone, mesalazine	Active UC	Colon descendens	0.8057	1.1579
UC 4	M	53	Infliximab, prednisolone	Active UC	Colon descendens	0.5482	0.9525
UC 5	M	41	Vedolizumab	Active UC	Colon sigmoideum	0.1410	0.0740
UC 6	M	59	Adalimumab, prednisolone	Chronic UC	Colon descendens	2.1653	1.0526
UC 7	M	55	Mesalazine	Chronic UC Colitis-associated carcinoma	Colon descendens	1.2074	1.2264
UC 8	F	20	None	Indeterminate colitis	Colon descendens	0.7710	0.7946
UC 9	M	60	Prednisolone, mesalazine	Chronic UC	Colon descendens	1.0324	0.9578
UC 10	F	38	None	Chronic UC Colitis-associated carcinoma Colon	Descendens	0.8791	0.9460
UC 11	F	60	Infliximab	Vezolizumab	Chronic UC Colon descendens	0.4683	1.0890

*IEN, intraepithelial neoplasia. Pixel intensities of arpin and ArpC5 bands (normalized to their respective actin bands) from blots in Figure 4B are shown.*

### Statistics

Data are presented as mean ± standard deviation (SD) and are representative of at least three independent experiments. Significance between groups was assessed by two-tailed Student’s *t*-test, two-tailed *t*-test followed by a Mann–Whitney test, or ANOVA with Bonferroni’s correction or Tukey’s *post hoc* test, as indicated in the figure legends. Statistical analyses were performed using GraphPad Prism software v5.0. Values of probability p < 0.05 were considered statistically significant. ^∗^*p* < 0.05; ^∗∗^*p* < 0.01; ^∗∗∗^*p* < 0.001.

## Results

### Arpin Is Expressed in Epithelium and Associates With Junction Proteins

**FIGURE 1:**
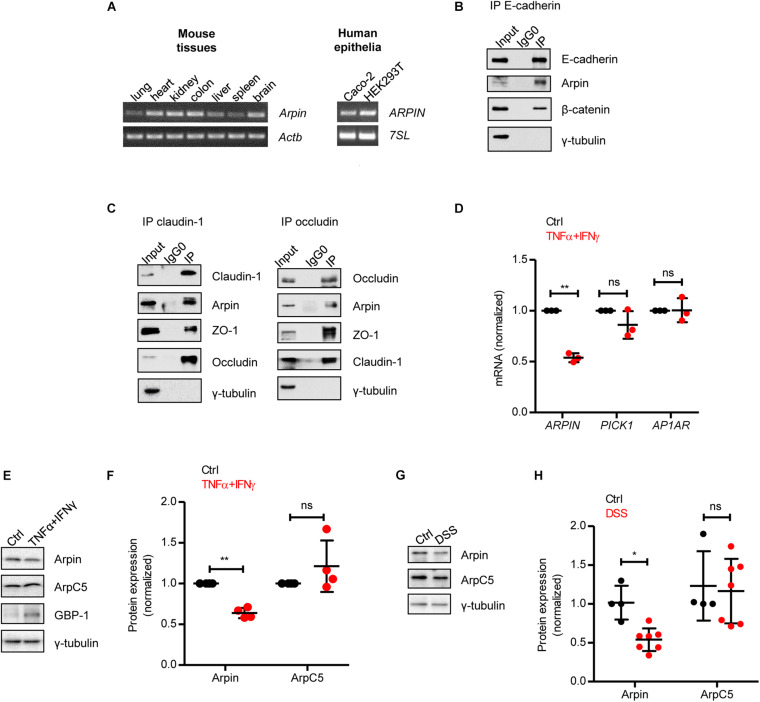
Arpin is downregulated under inflammatory conditions. **(A)** RT-PCR for *Arpin* and *Actb* using cDNA derived from the indicated mouse tissues (left) and *ARPIN* and *7SL* as housekeeping gene using cDNA derived from the indicated human cell lines (right); *n* = 3. **(B)** Western blot of E-cadherin immunoprecipitates. β-catenin was probed as a positive control of interaction and γ-tubulin as a negative control; *n* = 3. Input = whole cell lysate; IgG0 = IP using IgG as control, IP = IP using specific antibodies. **(C)** Western blots of claudin-1 and occludin immunoprecipitates. ZO-1 was probed as a positive control and γ-tubulin as a negative control, *n* = 3. **(D)** Quantitative real-time RT-PCR for the Arp2/3 inhibitors *ARPIN*, *PICK1*, and *AP1AR* using cDNA from Caco-2 colon epithelial cells treated or not with tumor necrosis factor (TNF)α/interferon (IFN)γ (*n* = 3; two-tailed *t*-test). Data are shown as relative expression normalized to the housekeeping gene *7SL*. **(E)** Western blot for arpin and the Arp2/3 subunit ArpC5 in Caco-2 cells treated or not with TNFα/IFNγ. Guanylate-binding protein-1 (GBP-1) was used as an inflammation positive control. **(F)** Densitometric analysis of panel **(E)** (*n* = 4; two-tailed *t*-test). **(G)** Western blot for arpin and ArpC5 in colons from control and dextran sulphate sodium (DSS)-treated mice. **(H)** Densitometric analysis of panel **G** (nCtrl = 4, nDSS = 7; two-tailed *t*-test followed by Mann–Whitney test). ns, non-significant; *p < 0.05; **p < 0.01.

Arpin is a recently discovered PIA, and little is known regarding its expression profile in organs and tissues. We performed RT-PCR analysis and observed arpin mRNA expression in mouse lung, heart, kidney, liver, spleen, brain, and colon (Figure 1A). To explore the potential role of arpin in colon epithelium, we confirmed arpin expression in monolayers of the well-established human colon epithelial cell line Caco-2 by RT-PCR (Figure 1A). Arpin expression was also observed in monolayers of the epithelial cell line HEK293 (Figure 1A). The integrity and function of the intestinal epithelial barrier (IEB) significantly rely on the stability of epithelial junctions. We analyzed a publicly available dataset of E-cadherin’s interactome in epithelial cells and found arpin as a potential binding partner of E-cadherin (Guo et al., 2014). Thus, we performed immunoprecipitation assays and found that arpin co-precipitated not only with the AJ protein E-cadherin (Figure 1B) but also with the TJ proteins claudin-1 and occludin (Figure 1C), suggesting that arpin is located at junctions and involved in IEB regulation. It remains to be proven whether these interactions with transmembrane proteins are direct or mediated by the scaffold proteins β-catenin and zonula occludens 1 (ZO-1) that are known interaction partners of E-cadherin and claudin-1/occludin, respectively. Tubulin was used as a negative control and absent in all precipitates (Figures 1B,C). In an effort to search for potential functional domains, we performed an *in silico* analysis of the arpin protein sequence using “The Eukaryotic Linear Motif” and “PhosphoSitePlus” resources (Hornbeck et al., 2012; Kumar et al., 2020). Several putative interaction motifs and three putative phosphorylation residues were found that remain to be experimentally confirmed (Supplementary Figure 2).

### Arpin Is Downregulated During Inflammation Both *in vitro* and *in vivo*

The pro-inflammatory cytokines TNFα and IFNγ synergize to induce IEB dysfunction (Wang et al., 2005; Capaldo et al., 2014). In order to unravel the expression of PIA during inflammation, we analyzed the expression of *ARPIN*, *PICK1*, and *AP1AR* (human homolog of gadkin) in control and TNFα/IFNγ-treated Caco-2 cells by qRT-PCR and found that only arpin was significantly downregulated during inflammation (Figure 1D). Western blots and subsequent densitometric analysis confirmed this decrease at the protein level (Figures 1E,F). Importantly, the ArpC5 subunit (representative of Arp2/3) showed no significant changes, and guanylate-binding protein-1 (GBP-1), a marker for IFN responses used here as a positive control for inflammation, was upregulated (Figure 1E). Of note, arpin protein was also significantly downregulated in colons from mice with DSS-induced colitis, whereas ArpC5 levels were again unaltered (Figures 1G,H). Disease parameters proving the severity of colitis in these experiments are shown in Supplementary Figure 3. We also analyzed arpin expression in the ileum (the most proximal part of the small intestine to the colon), in which also inflammation in response to DSS has been described (Rehal et al., 2018). However, we did not observe significant differences in arpin levels in DSS-treated ileun in comparison to controls (Supplementary Figure 4). It remains to be determined whether the reduction in arpin levels is indeed specific to colon inflammation. In future studies, we will investigate arpin functions in other inflammation models.

### Arpin Depletion Increases Epithelial Permeability and Actin Filament Formation and Causes Junction Disruption

**FIGURE 2:**
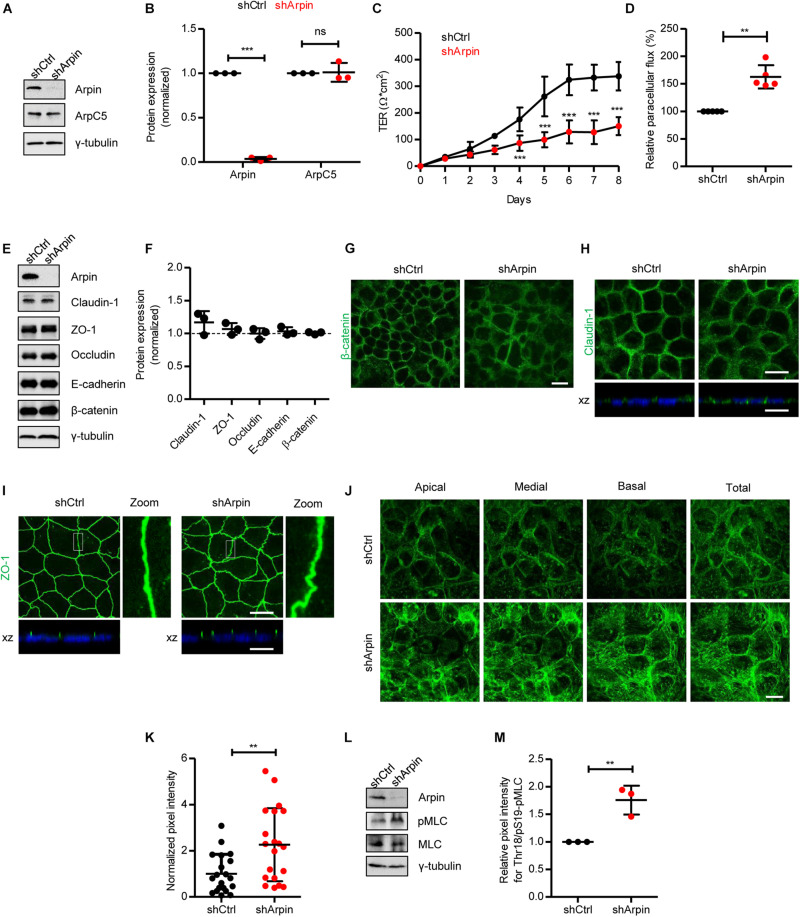
Arpin depletion causes hyperpermeability and altered architecture of junctions and the actin cytoskeleton. **(A)** Western blot for arpin and ArpC5 of lysates from control (shCtrl) and arpin-depleted (shArpin) Caco-2 cells. **(B)** Densitometric analysis of panel **(A)** (*n* = 3; two-tailed *t*-test). **(C)** Transepithelial electrical resistance (TER) development in control and arpin-depleted Caco-2 monolayers (*n* = 6; two-way ANOVA with Bonferroni‘s correction). **(D)** Paracellular flux using confluent control and arpin-depleted Caco-2 monolayers (*n* = 3; two-tailed *t*-test). **(E)** Western blot for claudin-1, zonula occludens 1 (ZO-1), occludin, E-cadherin, and β-catenin in control and arpin-depleted cells. **(F)** Densitometric analysis of panel **E** (*n* = 3; two-tailed *t*-test). **(G)** Immunostaining for β-catenin (green) in control and arpin-depleted Caco-2 monolayers (*n* = 3). Bar = 20 μm. **(H)** Immunostaining for claudin-1 in control and arpin-depleted Caco-2 monolayers. xz-planes are shown below for claudin-1 (green) and the nuclei as reference (blue). Images are representative of *n* = 3. Bar = 20 μm. **(I)** Immunostaining for ZO-1 (green) in control and arpin-depleted Caco-2 monolayers. xz-planes are shown below with nuclei as reference (blue); *n* = 3. 3 × digital zoom of junctions is shown on the right. Bar = 20 μm. **(J)** Location of apical, medial, and basal actin filaments in control and arpin-depleted Caco-2 cells stained with phalloidin; *n* = 4. Bar = 20 μm. **(K)** Actin density quantification normalized to the average of shCtrl cells (*n* = 20 cells per condition randomly selected from four independent experiments, two-tailed *t*-test). **(L)** Western blot for myosin-II light chain (MLC) and pMLC in control and arpin-depleted cells. **(M)** Densitometric analysis of panel **(L)** (*n* = 3; two-tailed *t*-test). **p < 0.01; ***p < 0.01. ns = not significant.

To analyze the role of arpin in IEB regulation, we generated stable arpin-depleted Caco-2 cells with 96% reduction in arpin protein levels (Figures 2A,B). These cells did not show alterations in total levels of ArpC5 (Figures 2A,B). Interestingly, although monolayer formation was achieved, loss of arpin caused a significant decrease in TER (Figure 2C), along with increased macromolecular permeability for 4 kDa FITC-dextran (Figure 2D). We then tested whether such a decrease in TER was associated with an alteration of junctional proteins. Protein levels of ZO-1, occludin, claudin-1, E-cadherin, and β-catenin showed no changes in arpin-depleted cells when compared to control cells (Figures 2E,F). By contrast, we did observe changes in the distribution pattern of β-catenin, claudin-1, and ZO-1. Control cells showed the expected mostly junctional localization of β-catenin, whereas arpin-depleted Caco-2 cells showed predominantly cytosolic staining of β-catenin (Figure 2G), suggesting that the absence of arpin favors internalization of β-catenin. Given the importance of AJ for intercellular epithelial adhesion, it seems likely that disturbed AJ composition affects TJ architecture. In control cells, claudin-1 was detected at the periphery and apical regions of cells in the monolayer (Figure 2H), whereas in arpin-depleted cells, the claudin-1 signal showed a more diffuse pattern at the cell periphery and partial loss of apical localization, suggesting that claudin-1 gets more internalized without arpin (Figure 2H). On the other hand, the apical localization of ZO-1 was preserved at cell contacts. However, we observed morphological differences in ZO-1 patterns, changing from strictly linear in control cells to wavy in arpin-depleted cells (Figure 2I). Such wavy ZO-1 patterns have previously been reported to be correlated with increased epithelial permeability (Zhou et al., 2013) and are thus in agreement with our permeability data described above. On the other hand, the apical junctional actin belt is critical for epithelial barrier integrity, and it is intuitive to think that arpin as Arp2/3 inhibitor would affect actin dynamics. Indeed, F-actin labeling using phalloidin showed an overall increased actin filament content in arpin-depleted Caco-2 cells (Figure 2J). Quantification of the pixel intensity of the phalloidin signal revealed a statistically significant increase in F-actin content in the absence of arpin (Figure 2K). This result is in agreement with the fact that arpin is an Arp2/3 inhibitor, and its absence would lead to more active Arp2/3 and thus increased formation of Arp2/3-dependent branched actin filaments (Verma et al., 2004; Dang et al., 2013). However, in addition to increased cortical F-actin, we also observed increased fibers crossing the cell body resembling contractile stress fibers that are known to exert pulling forces on junctions and thus contribute to junction destabilization (Bogatcheva and Verin, 2008, 2009; Citalan-Madrid et al., 2017; He et al., 2020). Increased contractility of the perijunctional actomyosin ring leading to reorganization of tight junction and increased epithelial permeability is a consequence of myosin-II light chain kinase (MLCK) and Rho-associated coiled-coil containing protein kinase 1 (ROCK1) activation and myosin-II light chain (MLC) phosphorylation. Indeed, we detected a significant increase in MLC phosphorylation (Figures 2L,M), suggesting that this is the mechanism driving junction destabilization in the absence of arpin. During intestinal inflammation, this mechanism is triggered by pro-inflammatory cytokines including TNFα and IFNγ (Bruewer et al., 2003; Lechuga and Ivanov, 2021). Given that arpin is clearly downregulated by TNFα and IFNγ (Figures 1D–H), arpin can be considered part of the molecular machinery driving inflammation-induced actin remodeling and junction disruption.

### Arpin Depletion Alters Epithelial Morphology

**FIGURE 3:**
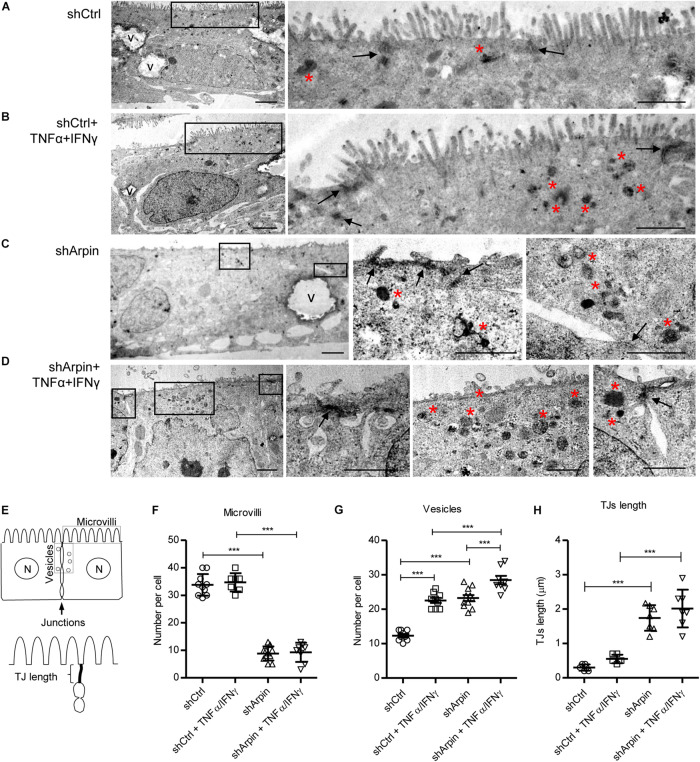
Arpin depletion alters the ultrastructure of intestinal epithelial cells. Morphology of resting **(A)** and tumor necrosis factor (TNF)α/interferon (IFN)γ-treated **(B)** control cells (shCtrl). Morphology of resting **(C)** and TNFα/IFNγ-treated **(D)** arpin-depleted cells (shArpin). In the low-magnification micrographs on the left, overall morphology of entire epithelial cells can be observed (V, vacuoles; bar = 2 μm). Frames indicate areas from which the high-magnification images (on the right) were taken. Arrows indicate tight junctions at the apical-most cell contacts. Red asterisks indicate accumulation of electron-dense endocytic vesicle (right images, bar = 1 μm). **(E)** Scheme of the method for quantification of the number of microvilli, vesicles, and tight junction (TJ) length. Panels **(F–H)** correspond to the quantification of number of microvilli, number of vesicles, and TJ length, respectively (*n* = 6–11; one-way ANOVA with *post hoc* Tukey test). ***p < 0.001.

To further support the concept that arpin regulates junction architecture and epithelial morphology, we performed ultrastructural analysis by transmission electron microscopy of control and arpin-depleted cells in the absence and presence of TNFα/IFNγ. Control cells showed normal morphology including homogeneously distributed microvilli along the apical surface (Figure 3A). As expected, control cells had discrete dense TJ with very few electron-dense structures corresponding to endocytic vesicles near them (Figure 3A). When control cells were exposed to TNFα/IFNγ, microvilli were mostly conserved, but several electron-dense vesicles were observed close to TJ likely corresponding to inflammation-induced internalization processes (Figure 3B). Surprisingly, in arpin-depleted cells, microvilli were mostly lost, and TJ appeared as elongated wavy electron-dense structures with several electron-dense vesicles in the TJ vicinity (Figure 3C). Arpin-depleted cells treated with TNFα/IFNγ showed many more electron-dense vesicles of different sizes near the TJ (Figure 3D). Quantification of microvilli, vesicles, and TJ lengths was performed according to the scheme in Figure 3E. The number of microvilli per cell was similar in control cells with and without TNFα/IFNγ. Absence of arpin strongly reduced the numbers of microvilli, and this was not further reduced by TNFα/IFNγ treatment (Figure 3F). Strikingly, the number of electron-dense vesicles close to TJ was significantly increased to the same extent by both arpin depletion or TNFα/IFNγ treatment (Figure 3G). Of note, TNFα/IFNγ-treated arpin-depleted cells showed even a higher number of vesicles surrounding junctions, suggesting that inflammatory stimuli can induce even more internalization in the absence of arpin. Arpin depletion, in contrast to TNFα/IFNγ, also caused significant elongation of TJ (Figure 3H), which is in agreement with the observed wavy ZO-1 pattern (Figure 2H). Taken together, these data show that arpin is a critical regulator of epithelial morphology, including microvilli integrity and TJ architecture. Loss of arpin alone induces an increase in junction-associated vesicles similar to pro-inflammatory stimuli, which is most likely the reason for the observed barrier dysfunction. Thus, we conclude that arpin localizes at junctions to maintain Arp2/3 activity low, thus preventing spontaneous internalization.

### Arpin Is Downregulated in Colon Tissue From Patients With Ulcerative Colitis

**FIGURE 4:**
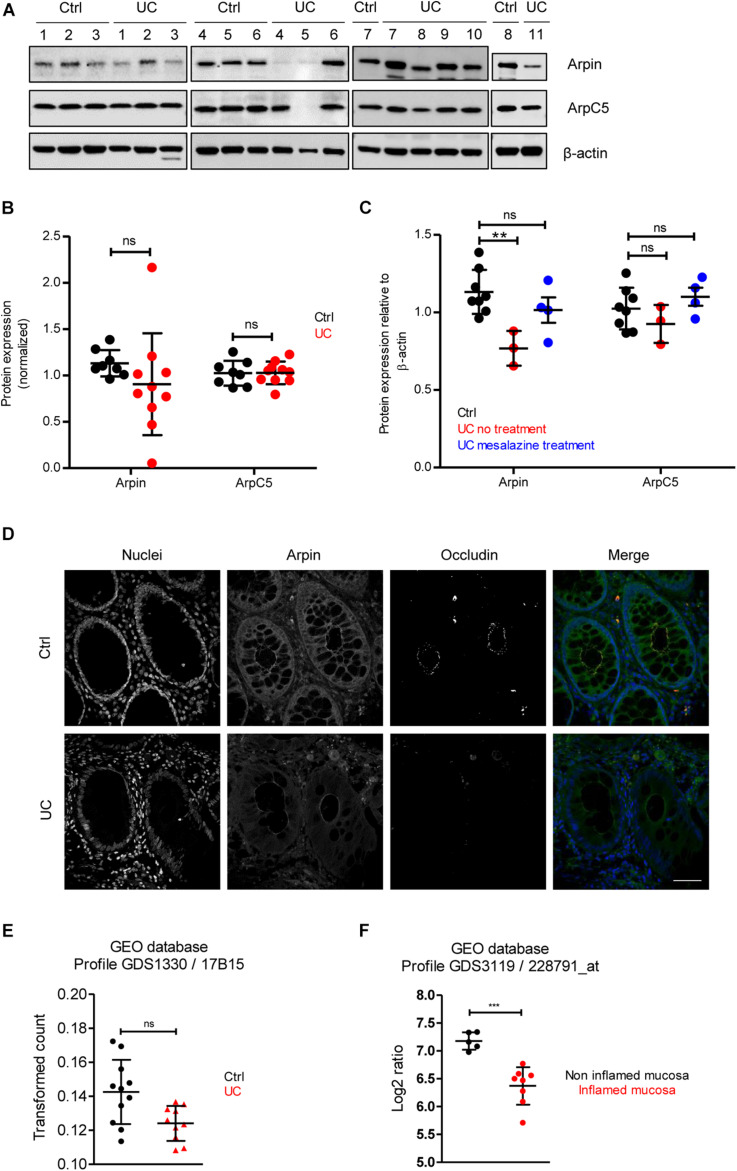
Arpin is downregulated in inflamed areas of colon tissue from ulcerative colitis (UC) patients. **(A)** Western blot for arpin and ArpC5 from resection specimens of patients with UC and non-inflamed controls. β-actin was probed as the loading control. **(B)** Quantification of pixel intensities including all bands (compare Table 1 for single values; nCtrl = 8, nUC = 10; two-tailed *t*-test followed by Mann–Whitney test; ns, non-significant. We only excluded sample UC 5 as a technical outlier because of the absence of both arpin and ArpC5 and a much weaker β-actin band. **(C)** Comparison of pixel intensities from bands of control samples, samples from patients receiving no treatment, and patients receiving mesalazine in their medication regimen. **p < 0.01. **(D)** Representative immunostaining for occludin and arpin from resection specimens of patients with histologically active UC and non-inflamed controls; *n* = 3. Bar = 20 μm. **(E)** Arpin mRNA analysis in biopsies from UC patients compared to controls (n: Control = 11, UC = 10; one-way ANOVA with Kruskal–Wallis correction. **(F)** mRNA analysis of arpin from publicly available datasets (n: non-inflamed mucosa = 5, inflamed mucosa = 8; two-tailed *t*-test with Welch’s correction). ***p < 0.001. ns = not significant.

To provide insights about the relevance of arpin expression in human disease, we analyzed arpin protein levels in the human inflammatory bowel disease UC, in which IEB functions are compromised (Martini et al., 2017). Patient characteristics are presented in Table 1. Western blot analyses of tissue samples from UC patients and patients undergoing colon surgery who did not suffer from inflammatory bowel diseases revealed that arpin was differently expressed in colon tissue samples of UC patients with a tendency toward lower levels compared to controls that did not reach statistical significance due to the high variation (Figures 4A,B). In agreement with our observations in cells and mice, ArpC5 levels were similar in control and UC tissues. While there was no clear correlation of arpin levels with patient characteristics (Table 1), it is interesting to note that arpin levels were significantly lower in those patients who did not take any medication compared to controls and that arpin levels were restored to normal levels in UC patients taking mesalazine in their medication regimen, suggesting that mesalazine can contribute to arpin restoration as a mechanism of alleviating inflammation (Figure 4C). Also, in these groups, ArpC5 levels were not significantly changed.

Arpin downregulation in tissue areas that showed histological signs of acute inflammation (crypt dysplasia, occludin downregulation) was confirmed by confocal microscopy (Figure 4D). Moreover, clear changes in arpin localization could be observed. In control tissue, arpin was clearly enriched in the cell periphery close to cell contacts along the basolateral border together with a sharp occludin signal. In UC tissue, the arpin signal along the basolateral border was lost and the signal was more diffusely distributed (Figure 4D). Next, we analyzed RNA sequencing (RNAseq) data from control and UC tissue biopsies available in the Gene Expression Omnibus (GEO) database. An overall comparison between control and UC tissues showed a trend toward lower arpin levels in UC tissue that was non-significant (Figure 4E). By contrast, comparing arpin expression in inflamed and non-inflamed areas of UC patients, a significant downregulation of arpin in the inflamed areas was revealed (Figure 4F). Thus, the RNAseq data clearly confirm our protein data that arpin is differently expressed and only reduced if the tissue is acutely inflamed. Together, these data show that loss of arpin could be a novel hallmark of acute inflammation in UC.

### CK666 Strengthens the Intestinal Epithelial Barrier and Ameliorates Tumor Necrosis Factor-α/Interferon-γ-Induced Epithelial Dysfunction *in vitro*

**FIGURE 5:**
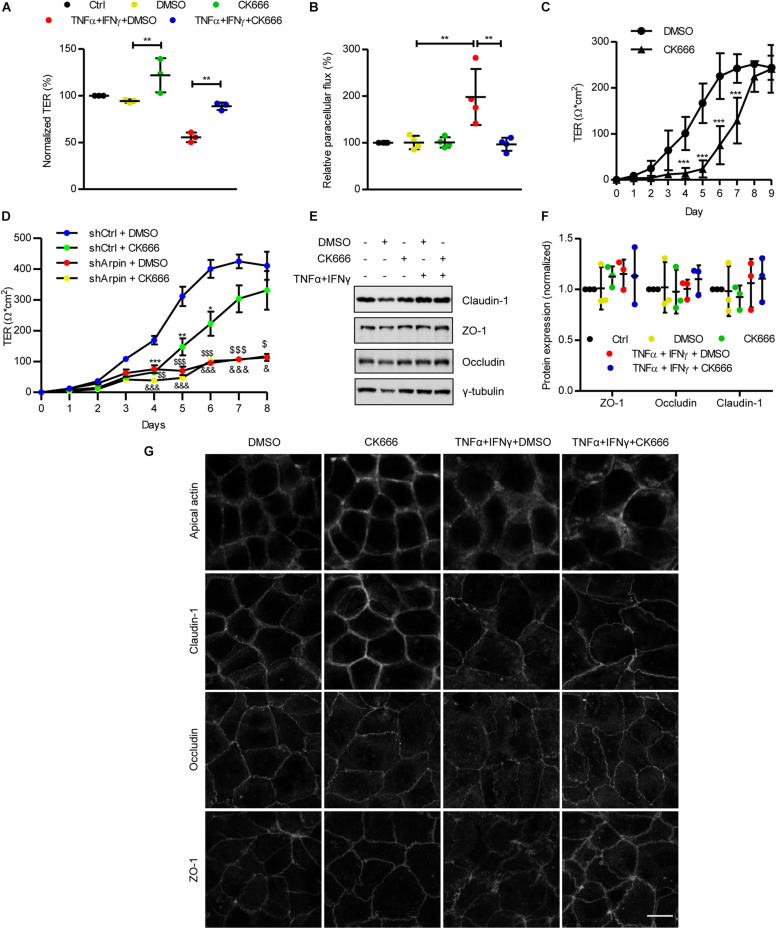
CK666 reinforces the epithelial barrier. **(A)** Transepithelial electrical resistance (TER) measurements of confluent Caco-2 cells treated or not for 48 h with tumor necrosis factor (TNF)α/interferon (IFN)γ and CK666. The vehicle dimethylsulfoxide (DMSO) was used as control (*n* = 3; one-way ANOVA). **(B)** Paracellular flux of confluent Caco-2 cells treated or not for 48 h with TNFα/IFNγ and CK666 was measured 2 h after adding 4 kDa fluorescein isothiocyanate (FITC)-dextran; color code as in panel **(A)**; *n* = 4; one-way ANOVA. **(C)** Time-course TER measurements of sparse Caco-2 cells in the presence or absence of CK666 (*n* = 5; two-way ANOVA). **(D)** Time-course TER measurements of sparse control and arpin-depleted Caco-2 cells in the presence or absence of CK666 (*n* = 6; two-way ANOVA). Data are compared with shCtrl DMSO-treated cells (*p vs. shCtrl + CK666, ^$^p vs. shArpin + DMSO, and ^&^p vs. shArpin + CK666). **(E)** Western blot for claudin-1, zonula occludens 1 (ZO-1), and occludin of Caco-2 monolayers treated or not with TNFα/IFNγ in the presence or absence of CK666. **(F)** Densitometry analysis of panel **(D)** (*n* = 3; one-way ANOVA with Bonferroni’s correction). **(G)** Confocal microscopy analysis of apical actin, claudin-1, occludin, and ZO-1 in Caco-2 cells treated or not with TNFα/IFNγ and CK666; *n* = 3. Bar = 10 μm. **p < 0.01; ***p < 0.001.

To date, arpin’s functions have been attributed exclusively to its role as an Arp2/3 inhibitor. Given that the depletion of arpin (i.e., decrease in Arp2/3 inhibition) leads to alteration of the IEB, as shown by permeability assays (Figures 2C,D), we tested whether pharmacological Arp2/3 inhibition would also regulate IEB integrity. We analyzed the effect of the Arp2/3-specific small-molecule inhibitor CK666 on IEB functions under basal and inflammatory conditions. Caco-2 epithelial monolayers were treated with CK666 or vehicle (DMSO) in the presence or absence of TNFα/IFNγ. As expected, a significant reduction in TER (Figure 5A) and an increase in paracellular flux of 4 kDa FITC-dextran (Figure 5B) were observed after treatment with TNFα/IFNγ compared to control cells. Interestingly, co-treatment with CK666 significantly ameliorated cytokine-induced permeability (Figures 5A,B). Surprisingly, CK666 alone increased epithelial barrier integrity, as indicated by an increase in TER of control monolayers (Figure 5A), whereas it did not affect paracellular flux under basal conditions (Figure 5B). These results demonstrate for the first time that Arp2/3 inhibition protects established epithelial monolayers and attenuates IEB dysfunction under inflammatory conditions *in vitro*. We emphasize that this protective effect of CK666 was only observed in well-established cell monolayers because treatment of sparse Caco-2 cells with CK666 significantly delayed formation of monolayers (Figure 5C), confirming published data that Arp2/3 is needed for the formation of monolayers likely because it also regulates proliferation and lamellipodia formation required for cell migration and establishing cell contacts (Henson et al., 2015; Molinie et al., 2019). Next, we asked whether the observed effects on TER development in the absence of arpin and with CK666 treatment are related. To this end, we performed experiments in which we treated arpin-depleted Caco-2 cells with the specific Arp2/3 inhibitor CK666 and monitored TER development. Interestingly, Arp2/3 inhibition with CK666 did not show additional effects on top of the effects of arpin depletion (Figure 5D), suggesting that potential Arp2/3 effects on junction formation are fully reflected by the arpin knockdown. It will be interesting to analyze in more detail the relation of arpin and Arp2/3 in epithelial cells, as it was recently demonstrated for Arp2/3 and formin-like proteins (FMNL2/3) using Arp2/3-depleted cells (Dimchev et al., 2021).

### Tumor Necrosis Factor-α/Interferon-γ-Induced Internalization of Junction Proteins Is Prevented by CK666

While total junction protein levels are usually unchanged in epithelial monolayers challenged with TNFα/IFNγ, they are redistributed from junctions to the cytosol by endocytotic processes, causing barrier disruption and hyperpermeability (Li et al., 2008; Cao et al., 2013). In agreement with this, overall protein levels of the TJ proteins claudin-1, ZO-1, and occludin were not altered after cytokine exposure (Figures 5E,F). Neither CK666 alone nor in combination with TNFα and IFNγ changed these protein levels (Figures 5E,F), indicating that Arp2/3 inhibition does not induce changes in overall junction protein expression. Analysis of the distribution of these proteins by confocal microscopy revealed the expected internalization of claudin-1, ZO-1, and occludin after TNFα/IFNγ exposure. Interestingly, such internalization was ameliorated by co-treatment with CK666 (Figure 5G). In agreement with the TER increase (Figure 5A), CK666 alone led to accumulation of claudin-1 at cell contacts, especially at tricellular junctions (Figure 5G). TNFα/IFNγ-induced actin remodeling, as indicated by reduced apical actin rings, was also attenuated by CK666 (Figure 5G). These data show that inhibition of Arp2/3 strengthens IEB integrity of intestinal epithelial monolayers by maintaining junction and actin cytoskeletal architecture under basal and inflammatory conditions. These Arp2/3 inhibition data also agree with our arpin depletion data (theoretically more active Arp2/3) showing barrier dysfunction due to increased junction protein internalization.

### CK666 Protects Against Dextran Sulphate Sodium-Induced Colon Tissue Damage

**FIGURE 6:**
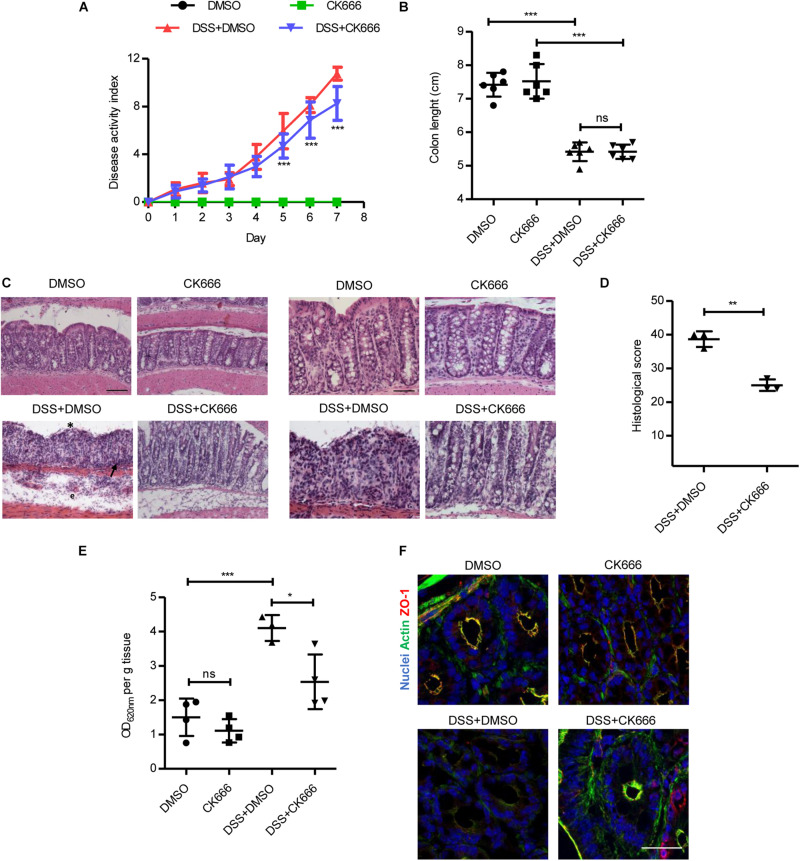
CK666 ameliorates tissue damage in a dextran sulphate sodium (DSS)-induced colitis mouse model. **(A)** Disease activity index of mice with DSS-induced colitis and treated with either the vehicle dimethylsulfoxide (DMSO) or 5 mg/kg CK666 intraperitoneally (i.p.) starting on day 3 (*n* = 8–9 per group; two-way ANOVA with Bonferroni’s correction). **(B)** Colon lengths after DSS-induced colitis (*n* = 6 per group; one-way ANOVA with Bonferroni’s correction). **(C)** Hematoxylin/eosin-stained colon tissue sections in 20 × magnification (left) and 40 × (right). Bars = 50 μm (left), 25 μm (right). Crypt dysplasia and ulceration (*), immune cell infiltration (arrow), and edema formation (e) are highlighted; *n* = 3 per group. **(D)** Histological inflammation score of colitic mice (*n* = 3 per group; two-tailed test). **(E)** Colon permeability to Evans blue in control and colitic mice treated with either DMSO or CK666 (*n* = 4 per group; one way-ANOVA with Bonferroni’s correction). **(F)** Immunostaining of zonula occludens 1 (ZO-1) (red) and actin (green) in colon tissue from control and colitic mice treated with DMSO or CK666; *n* = 3. Bar = 35 μm. *p < 0.05; **p < 0.01; and ***p < 0.001. ns = not significant.

We assessed whether the protective effect of Arp2/3 inhibition during inflammation *in vitro* also occurs *in vivo*. To this end, we administered CK666 in mice with DSS-induced colitis. The DAI consisting of body weight loss, diarrhea, and intestinal bleeding was evaluated daily during an experimental period of 7 days. Either 5 mg/kg of CK666 or DMSO alone (vehicle control) were i.p. injected daily starting on day 3 of DSS treatment when colitis symptoms started to manifest. The dose of 5 mg/kg of CK666 showed beneficial effects in previous studies *in vivo* (Park et al., 2013; Li et al., 2018). We also tested daily i.p. administration of CK666 at 5 mg/kg and 10 mg/kg from day 0 (Supplementary Figure 5A), without observing superior effects. No alterations in DAI were observed in the control groups that received water and were injected with DMSO or CK666 (Figure 6A). The group treated with DSS and injected with DMSO showed a progressive increase of DAI, reaching a maximum of 10.75 ± 0.53 on day 7. Importantly, colitic mice that were treated with CK666 reached a maximum DAI of only 8.25 ± 1.41 (Figure 6A), with the strongest protective effects being observed on intestinal bleeding followed by weight loss and stool consistency (Supplementary Figures 5B–D). No changes were detected in colon lengths between CK666-treated and control groups (Figure 6B). Histological analyses showed normal mucosal tissue morphology in DMSO- and CK666-treated animals (Figure 6C, micrographs at 20× and 40×). As expected, DSS-treated mice showed typical signs of colitis such as apical erosion, edema formation, crypt shortening, and immune cell recruitment. Importantly, all these colitis signs were reduced in DSS-treated mice that received CK666 injections (Figure 6C). Histological score showed a significant protective effect of CK666 on DSS-induced tissue damage (Figure 6D). Detailed evaluation of histological parameters as previously published (Shukla et al., 2018) revealed a significant protective effect of CK666 on loss of goblet cells, cryptitis, lamina propria inflammation, epithelial erosion, crypt dropout, and architectural distortion, whereas there was only a tendency toward improved hyperemia and mucosal edema (Supplementary Figure 6). Evans blue-based intestinal epithelial permeability assays *in vivo* showed similar low permeability in control and CK666-treated mice (Figure 6E). DSS treatment induced the expected strong increase in permeability, which was significantly reduced in colitic mice treated with CK666 (Figure 6E). This protective effect could be attributed to better conserved junction and cytoskeletal architecture, as CK666 prevented DSS-induced ZO-1 gaps at TJ and loss of the cortical apical actin ring (Figure 6F).

Together, our data demonstrate that arpin expression and Arp2/3 inhibition protect against IEB dysfunction during inflammation both *in vitro* and *in vivo*.

## Discussion

**FIGURE 7:**
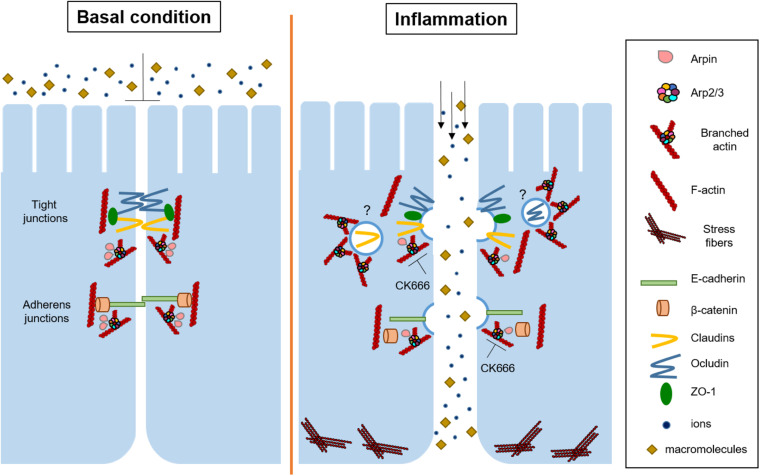
Current working model for arpin functions and Arp2/3 inhibition in intestinal epithelial cells. Under basal conditions, arpin localizes at cell junctions to maintain Arp2/3 activity low and prevent junction protein internalization. During inflammation, arpin is downregulated, allowing for Arp2/3 activation, junction protein internalization, stress fiber formation, and thus barrier dysfunction. On the other hand, CK666 strengthens the epithelial barrier and protects from inflammation-induced barrier dysfunction by preventing Arp2/3-dependent junction protein internalization. Question marks indicate that the exact mechanism of internalization is still unclear.

Here, we analyzed for the first time the role of the endogenous Arp2/3 inhibitory protein arpin in the regulation of IEB functions. Arpin, PICK1, and gadkin are endogenous PIAs that bind to Arp2/3 through their C-terminal acidic domain, and it has been suggested that they inhibit Arp2/3 in a compartmentalized manner within the cell (Molinie and Gautreau, 2018). Arpin is the most recently identified PIA that colocalizes with Arp2/3 and the Arp2/3 activator WAVE at lamellipodia (Dang et al., 2013). Because arpin is enriched at lamellipodia and these structures are involved in intercellular junction formation, we wondered whether arpin participates in tissue barrier regulation. The importance of both Arp2/3 and WAVE in cell–cell contact formation and permeability regulation in epithelial cells has been demonstrated (Verma et al., 2012; Zhou et al., 2013), but virtually nothing was known about the role of the endogenous negative regulation of Arp2/3 in this context. Here, we show that arpin is indeed ubiquitously expressed in mouse organs and epithelial cells and that arpin associates with the AJ proteins β-catenin and E-cadherin and the TJ proteins ZO-1, occludin, and claudin-1. Further experiments are needed to assess whether such interactions occur directly or through additional proteins. Of note, we now also show for the first time that after challenging colon epithelial cells with pro-inflammatory cytokines, only the mRNA levels of arpin were reduced, while mRNA expression of PICK1 and AP1AR (human gadkin homolog) remained unaltered, thus evidencing a relevant role of arpin during inflammation. Inflammation also caused downregulation of arpin protein *in vivo* in a DSS-induced colitis mouse model. Downregulation of arpin in this context likely means that less inhibitory protein is available at junctions to balance Arp2/3 activity, thus enabling Arp2/3 hyperactivation and Arp2/3-induced junction protein internalization and IEB disruption (Figure 7). This idea is supported by our findings that arpin-depleted cells had more internalized claudin-1 and β-catenin, lost linearity of ZO-1, had many vesicles close to elongated TJ, and showed increased epithelial permeability. These data agree with findings in ArpC3-deficient primary keratinocytes that also showed wavy ZO-1 patterns and hyperpermeability (Zhou et al., 2013). ZO-1 binds to the TJ proteins occludin and claudins and connects them to the actin cytoskeleton (Itoh et al., 1999; Fanning et al., 2007; Van Itallie et al., 2009, 2017), whereas β-catenin is involved in the connection of E-cadherin to the actin cytoskeleton (Buckley et al., 2014). Thus, it is also possible that arpin contributes to junction–actin anchorage and that its absence reduces junction stability, leading to epithelial barrier dysfunction. This is in line with the observed increased F-actin content and MLC phosphorylation, suggesting increased actomyosin contractility causing the observed junction destabilization. Whether these effects are a causal consequence of these altered actin dynamics or lack of direct arpin interaction with junction proteins or both needs to be unraveled in future studies.

The observation that arpin is reduced in many patients with UC suggests an important clinical relevance of arpin in the colon. Of note, arpin levels were closest to control levels in UC patients taking mesalazine within their medication regimen, indicating that loss of arpin is related to intestinal inflammation and that restoration of arpin expression by mesalazine may contribute to inflammation resolution. In this respect, it will be interesting to analyze possible functional relations of arpin with resolvins that participate in epithelial repair in UC. For example, resolvin E1 has recently been shown to trigger epithelial migration and proliferation and thus wound healing (Quiros et al., 2020). Arpin also controls proliferation because the Rac1/WAVE/arpin axis modulates G1/S cell cycle progression (Molinie et al., 2019). Arpin fine-tunes speed and persistence of cell migration (Dang et al., 2013) and induces pauses during migration that allow cells to turn (Gorelik and Gautreau, 2015), so that it may play a critical role during intestinal epithelial wound healing. Moreover, it is well known that chronic inflammation can lead to cancer development (Greten and Grivennikov, 2019). In particular, UC increases the risk to develop colorectal cancer (Rabbenou and Ullman, 2020). Arpin downregulation has been associated with the development of different cancer types, including colon cancer (Lomakina et al., 2016; Li T. et al., 2017; Li Y. et al., 2017; Zhang et al., 2018). Thus, a treatment that restores arpin expression, as shown here for mesalazine, is critical not only for UC treatment but also for colon cancer prevention. Our data that arpin is reconstituted in patients treated with mesalazine provide a possible mechanism of mesalazine action that is related to junction and actin functions mediated by arpin.

Very recently, arpin has also been shown to be essential for phagocytosis in macrophages (Jubrail et al., 2020). Thus, arpin seems to be a critical protein in different cell types for different cellular functions. We are only at the beginning of understanding the true physiological relevance of arpin, and certainly, more discoveries regarding arpin functions will emerge. An important question to address in the future is if and how arpin can also act independently of Arp2/3.

In our study, arpin functions correlated well with Arp2/3 inhibition, as arpin depletion (potential Arp2/3 activation) induced IEB dysfunction, whereas Arp2/3 inhibition improved it. Pharmacological inhibition of the Arp2/3 complex has been used as a tool to dissect biological functions of this complex. CK666 is a small molecule that binds to the Arp2/3 complex, stabilizes its inactive conformation, and blocks its activation without promoting disassembly of preformed actin branches (Nolen et al., 2009; Hetrick et al., 2013). Arp2 and ArpC2-depleted fibroblast treated with CK666 did not show additional phenotypic effects, highlighting the specificity of this compound (Wu et al., 2012).

We found that pharmacological blocking of Arp2/3 activity *via* CK666 during inflammation prevented barrier dysfunction triggered by pro-inflammatory cytokines. Considering that arpin depletion would contribute to Arp2/3 activation, all our data coincide well and point to arpin-mediated Arp2/3 inhibition to prevent junction protein internalization. However, the fact that inhibition of Arp2/3 by CK666 reinforced the epithelial barrier was surprising because other studies demonstrated that inhibition of Arp2/3 had detrimental effects on several functions such as migration, lamellipodia formation, and cytokinesis *in vitro* (Sun et al., 2011; Ilatovskaya et al., 2013; Henson et al., 2015). Moreover, under basal conditions in Madin-Darby canine kidney (MDCK) epithelial cell monolayers, CK666-mediated Arp2/3 inhibition resulted in increased paracellular flux to 3 kDa FITC-dextran (Van Itallie et al., 2015). On the other hand, in epithelial cells from *Drosophila* notum, Arp2/3 is required for E-cadherin internalization and regulation of junction stability (Georgiou et al., 2008). More research is needed to resolve these discrepancies, but they could be a result of different cell types, duration of treatment, and different cell confluency. At mature epithelial cell contacts, Arp2/3 activity is low (Sumida and Yamada, 2015) to only regulate the low basal physiological intercellular junction turnover. Arpin expression at epithelial cell contacts may thus be required to maintain this low Arp2/3 activity. In turn, loss of arpin in response to inflammation may then be required to allow for Arp2/3 activation to trigger the well-known inflammation-induced junction protein internalization. This interpretation is also in line with our data that Arp2/3 inhibition prevents junction protein internalization and reinforces the epithelial barrier.

The situation is different in a developing monolayer where there are no or few stable junctions. Here, inhibition of Arp2/3 rather affects proliferation and lamellipodia formation (Henson et al., 2015; Molinie et al., 2019) to delay monolayer formation, as confirmed here. This seems logical considering the fact that Arp2/3-dependent lamellipodia contribute to AJ assembly *via* clustering and assembly of E-cadherin plaques between neighboring cells (Baum and Georgiou, 2011).

The physiological relevance of our findings was proven *in vivo*, where CK666 attenuated the DAI, histological damage, and intestinal epithelial hyperpermeability in mice with DSS-induced colitis. Although it is important to consider that other cell types (e.g., immune cells) might be contributing to the observed effect on the intestinal epithelium due to the systemic delivery of CK666, our *in vitro* data support that indeed Arp2/3 inhibition in the epithelial cells is at least partly responsible for the IEB protection under inflammatory conditions. The extent of contribution of Arp2/3 inhibition in other cell types remains to be determined.

Arp2/3 is critical for proper organ functionality and development. For example, mice with specific deletion of Arp2/3 in the intestinal epithelium and the epidermis presented impaired transcytosis and dehydration (increased permeability), respectively, both resulting in lethality (Zhou et al., 2013, 2015). Thus, Arp2/3 is important in different contexts, but the same as with so many other proteins, its activity has to be controlled strictly for proper cell functions, and arpin might be the endogenous regulator providing this spatiotemporal control of activation levels in the intestinal epithelium.

## Data Availability Statement

The original contributions presented in the study are included in the article/Supplementary Material, further inquiries can be directed to the corresponding author.

## Ethics Statement

The studies involving human participants were reviewed and approved by Ethical Committee of the University of Würzburg (proposal numbers 113/13, 46/11, and 42/16) (61). The patients/participants provided their written informed consent to participate in this study. The animal study was reviewed and approved by Institutional Animal Care and Use Committee, CINVESTAVIPN.

## Author Contributions

SC-P performed the research and statistical analysis, analyzed and interpreted the data, and wrote the manuscript. AM-G, KC-O, JG-C, and HV-R performed the research and analyzed the data. RM-F performed the research and analyzed and interpreted the data. LC-B, MSh, and PN analyzed and interpreted the data. SF and NS analyzed human tissue samples and interpreted the data. AG provided essential reagents and interpreted the data. MSc conceived and supervised the study, analyzed and interpreted the data, and wrote the manuscript. All authors reviewed the manuscript.

## Conflict of Interest

The authors declare that the research was conducted in the absence of any commercial or financial relationships that could be construed as a potential conflict of interest.

## Supplementary Material

Supplementary Figure 1 Shows full blots including molecular weight markers.

Supplementary Figure 2 Shows the *in silico* analysis of arpin protein.

Supplementary Figure 3 Shows the disease activity index and colon length of colitic mice used to analyze arpin expression.

Supplementary Figure 4 Shows representative Western blots of ileum from control and DSS-treated mice and the quantification of normalized pixel intensities.

Supplementary Figure 5 Shows the disease activity index in mice treated with 5 and 10 mg/kg Ck666 daily and separated scores of the DAI presented in Figure 6A.

Supplementary Figure 6 Shows a detailed histological examination of colon tissues from colitic mice treated with Ck666 or vehicle.

Supplementary Table 1 Lists all primers.

Supplementary Table 2 Includes all primary and secondary antibodies.
