# Supplementary material for: The Arp2/3 Inhibitory Protein Arpin Is Required for Intestinal Epithelial Barrier Integrity
Source: Front Cell Dev Biol. 2021 May 3;9:625719. doi: 10.3389/fcell.2021.625719 (PMC8128147; doi:10.3389/fcell.2021.625719)
Supplement: Supplementary Figure 1 — Shows full blots including molecular weight markers. [file Data_Sheet_1.PDF]

Supplementary material

Table S1. Primer sequences.

**Gene**	**Forward 5'- 3'**	**Reverse 5'- 3'**
h7SL	ATCGGGTGTCCGCACTAAGTT	CAGCACGGGAGTTTTGACCT
hARPIN	GCCCAGAGTCACACAGCTAA	CTTTCTGAAGGGCAAGGAAG
hPICK1	ATGATTCAGGAGGTGAAGGG	CGGTGCTTGACTTTCTTCAA
hAP1AR	GAAATGACGACAGCACATCC	TAAGTGCTGCCCGTAGAATG
mArpin	GCTCTCTGTCAATTCCAGCA	CACCAGGAAGGTGAACACAG
mActb	TATCCACCTTCCAGCAGATGT	AGCTCAGTAACAGTCCGCCTA

Table S2. List of antibodies.

**Primary antibodies and phalloidin**						
Trademark	Catalog number	Specie	Target	Dilution for WB	Dilution for IF	Amount for IP
Thermo F. Scientific	T6557 (GTU-00)	Mouse	γ-tubulin	1:4000		
Santa Cruz	sc-7870 (H108)	Rabbit	E-cadherin	1:1000	1:50	2 μg
Santa Cruz	sc-7963 (E-5)	Mouse	β-catenin	1:500	1:50	
Invitrogen	61-7300	Rabbit	ZO-1	1:200	1:100	
Thermo F. Scientific	71-7800 (MH25)	Rabbit	Claudin-1	1:500	1:50	2 μg
Thermo F. Scientific	PA5-20755	Rabbit	Occludin	1:500	1:100	
Invitrogen	33-1500 (3F10)	Mouse	Occludin		1:100	2 μg
Santa Cruz	sc-53857 (1B1)	Rat	GBP-1	1:1000		
Sigma Aldrich	A3854	Mouse	β-Actin	1:10000		
Donated[1]	NA	Rabbit	arpin	1:500	1:50	
Biolegend	108402 (RB6-8C5)	Rat	Gr1		1:50	
Donated[2]	323H3	Mouse	ArpC5 (p16)	Undiluted	Undiluted	
Thermo F. Scientific	A12379	NA	AF-488 Phalloidin		1:200	
Cell Signaling	3672	Rabbit	MLC		1:500	
Cell Signaling	3674	Rabbit	pMLC (Thr18/Ser19)		1:500	

**Secondary antibodies**					
Trademark	Catalog number	Specie	Target	Dilution for WB	Dilution for IF
Santa Cruz	sc-2357	Mouse	Rabbit IgG-HRP	1:5000	
Santa Cruz	sc-2005	Goat	Mouse IgG-HRP	1:5000	
Santa Cruz	sc-2032	Goat	Rat IgG-HRP	1:5000	
Thermo F. Scientific	A11008	Goat	rabbit IgG-AF488		1:250
Thermo F. Scientific	A11001	Goat	Mouse IgG-AF488		1:250
Thermo F. Scientific	A11061	Rabbit	Mouse IgG, AF568		1:250

[1]Produced and donated by Dr. Alexis Gautreau (École Polytechnique, Paris, France), [2]Donated by Dr. Klemens Rottner and Dr. Theresia Stradal (Technical University Braunschweig, Helmholtz Centre for Infection Research, Braunschweig, Germany).
NA, Not applicable.

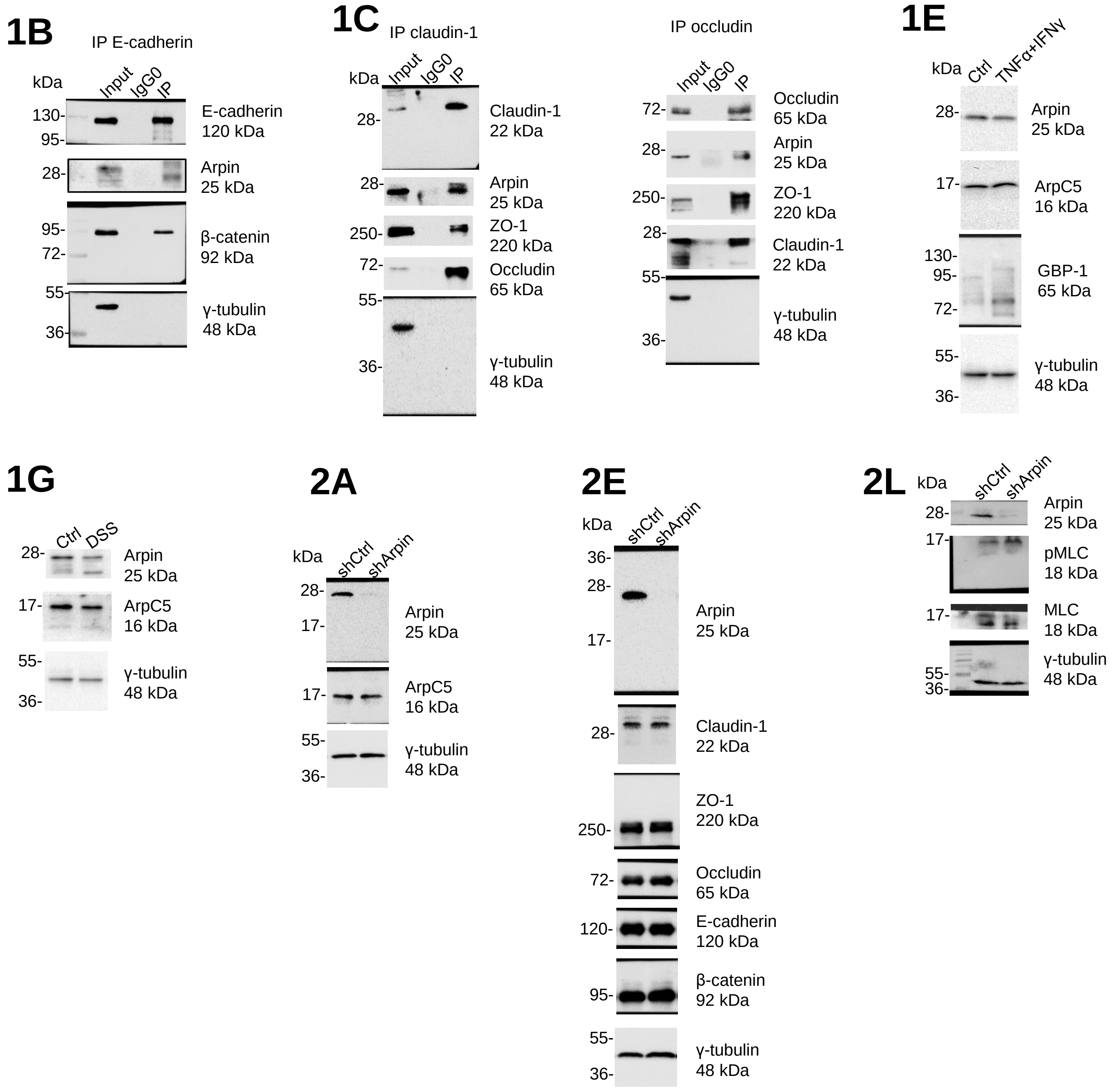

Figure S1. Western blots including molecular weight markers on membranes. Indicated figure numbers are those from the main figures to which the membranes belong. Please note, that we routinely cut membranes to incubate with tested, certified antibodies against proteins of different weights to have all relevant data from the very same membrane (excluding variations in transfer efficiency on different membranes).

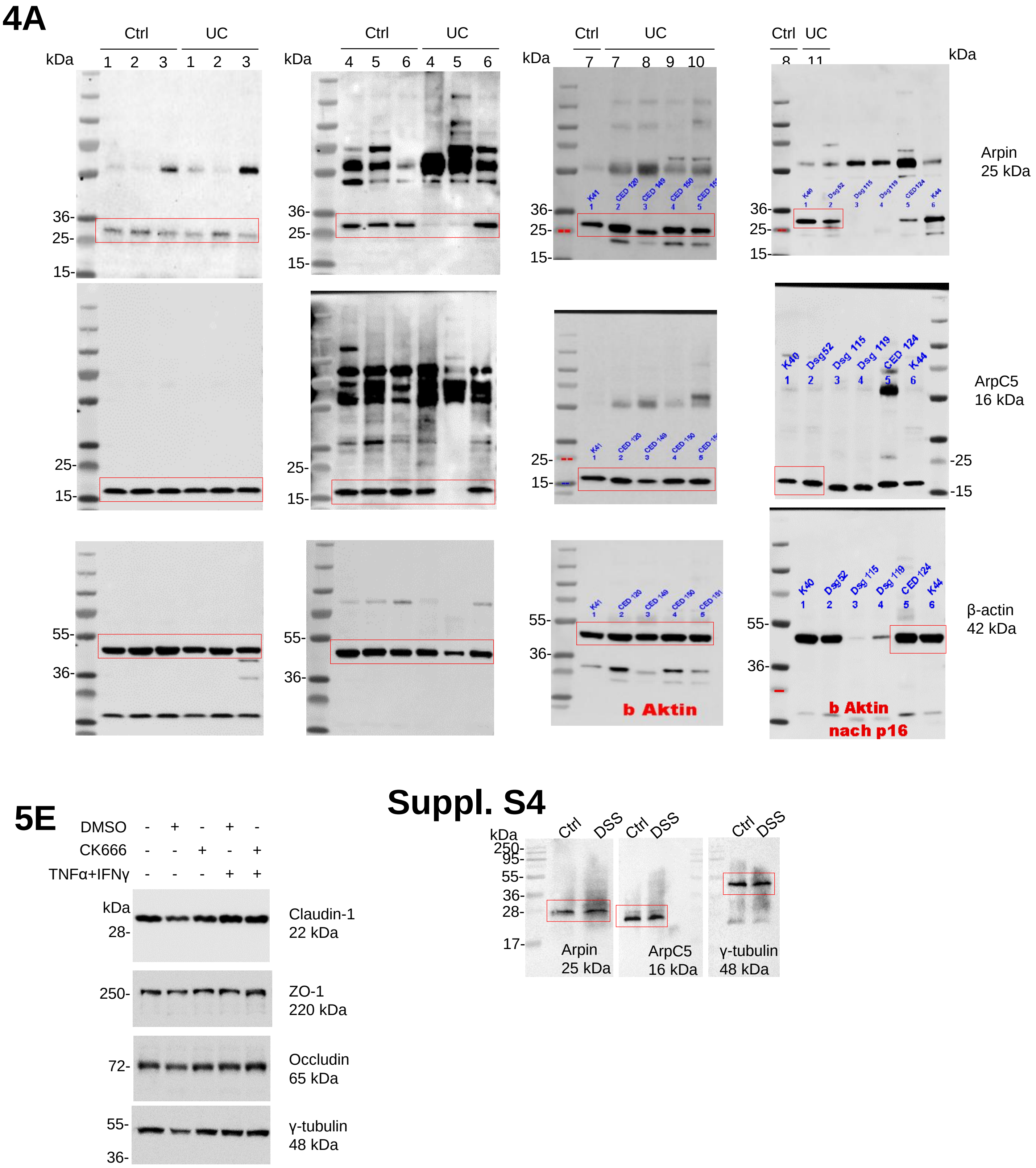

Figure S1 continued. Western blots including molecular weight markers on membranes. Indicated figure numbers are those from the main figures to which the membranes belong.

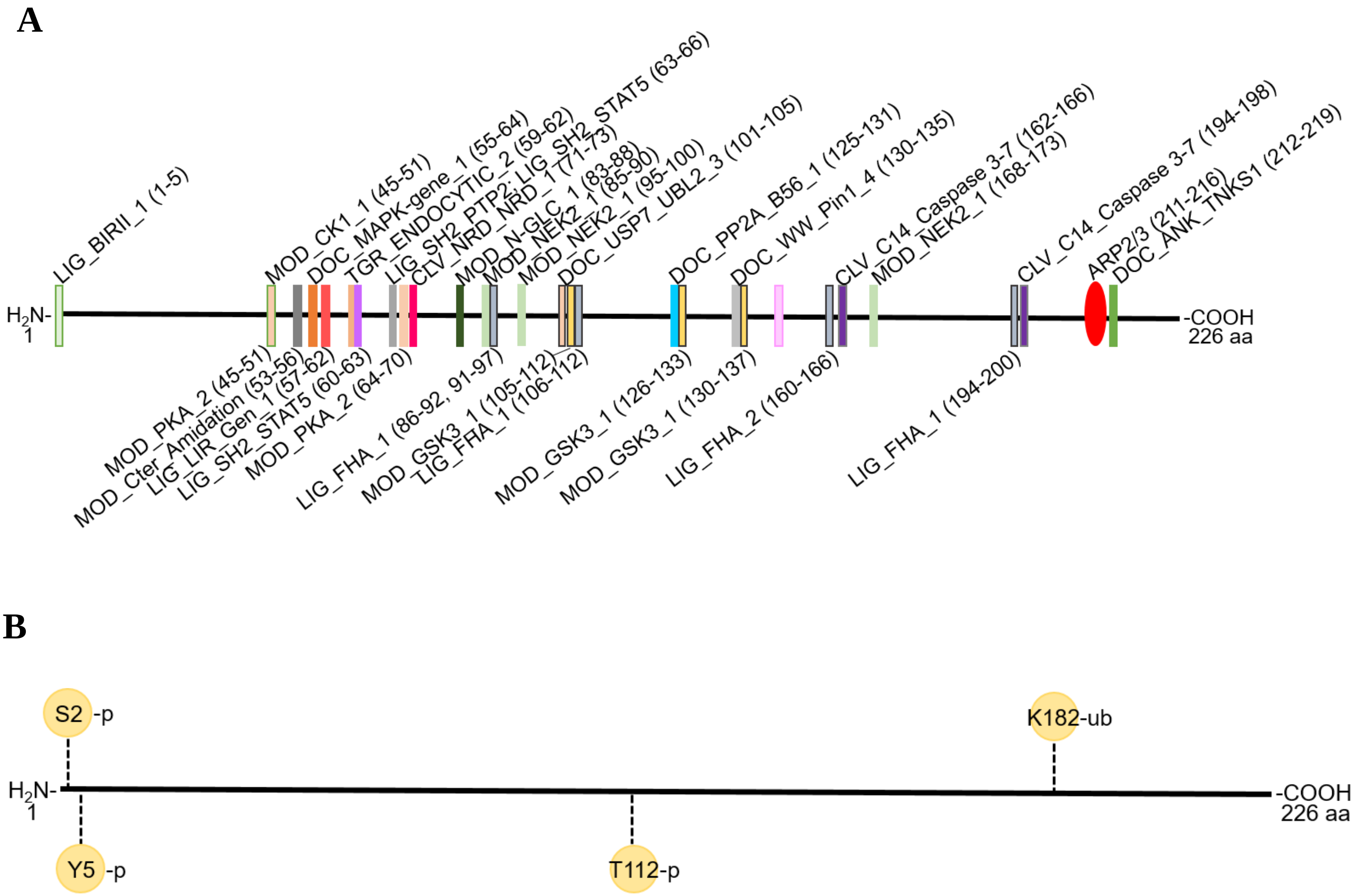

Figure S2. ***In silico*** **analysis of the arpin protein sequence. (A)** Bioinformatical analysis of the arpin protein sequence using "The Eukaryotic Linear Motif" resource (http://elm.eu.org/) showing interaction motifs. Motifs are functionally classified as ligand (LIG), targeting (TRG), docking (DOC), modification (MOD) or cleavage (CLV) motifs. Only highly conserved predicted motifs are represented. **(B)** Putative phosphorylation sites (p) and one ubiquitinylation site (ub) were identifed using the "PhosphoSitePlus" resource (https://www.phosphosite.org/homeAction.action). Only sites with at least 3 records discovered by mass spectrometry are shown.

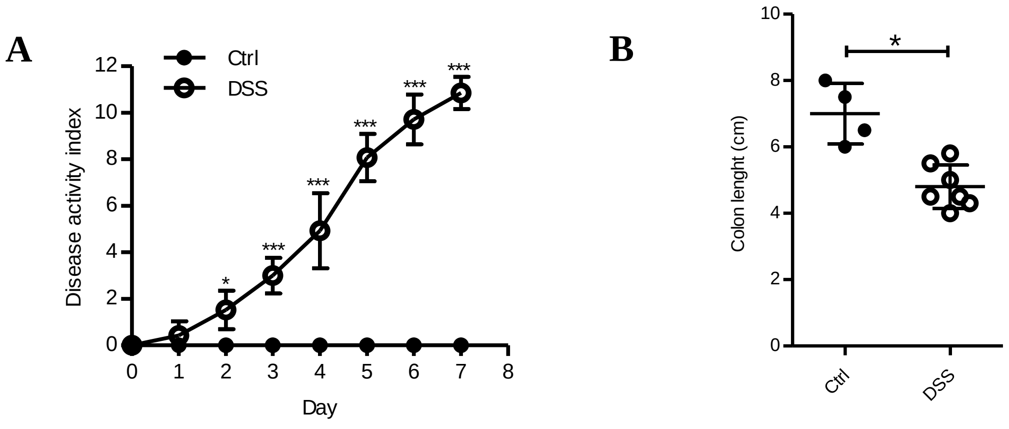

Figure S3. Characterization of the DSS colitis model. 7-day DSS colitis model corresponding to Fig. 1E: **(A)** Disease activity index (nCtrl=4, nDSS=7; 2-way ANOVA with Bonferroni‘s correction). **(B)** Colon lengths after DSS-induced colitis (nCtrl=4, nDSS=7; 2-tailed t-test with Welch‘s correction). $^{*}p<0.05$; $^{***}p<0.001$.

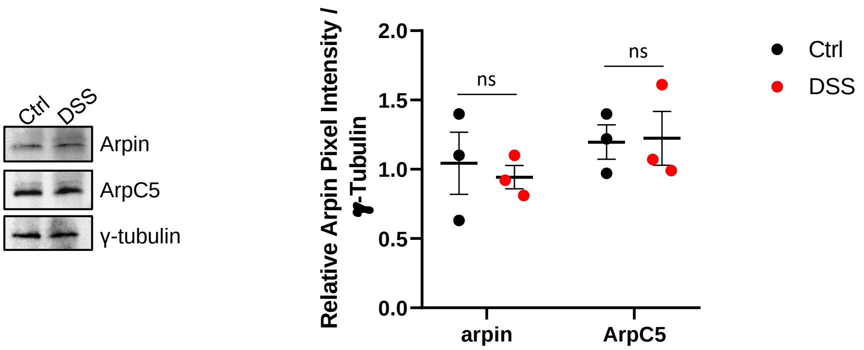

Figure S4. Arpin and ArpC5 protein levels are unaltered in the ileum of DSS-treated mice. (A) Representative Western blots of ileum from control and DSS-treated mice. **(B)** Quantification of relative pixel intensities normalized to tubulin levels (n=3; 2-tailed t-test with Welch's correction). ns, non-significant.

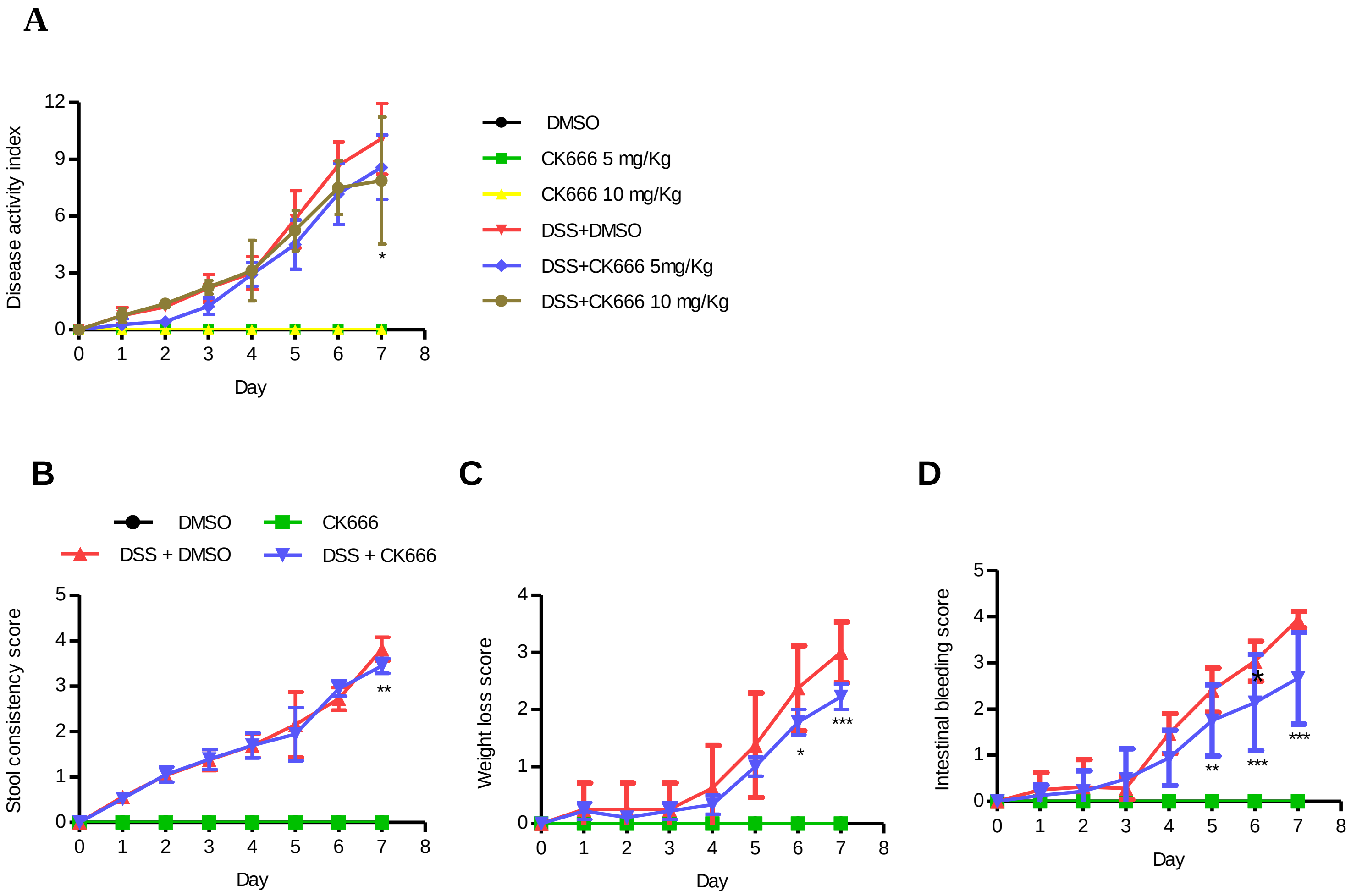

Figure S5. Disease activity index in colitic mice treated with CK666. Disease activity index of mice with DSS-induced colitis treated daily with the vehicle DMSO or CK666 at 5 and 10 mg/kg of weight (n=3 per group, except for DSS+CK666 10 mg/kg n=2; *p<0.05 DSS+DMSO vs DSS+CK666 10 mg/Kg; 2-way ANOVA with Bonferroni’s correction). **(B, C and D)** Separated scores corresponding to Fig. 6A: **(B)** stool consistency; **(C)** weight loss; and **(D)** intestinal bleeding. (n=8-9 per group; 2-way ANOVA with Bonferroni‘s correction). *p<0.05; **p<0.01; ***p<0.001

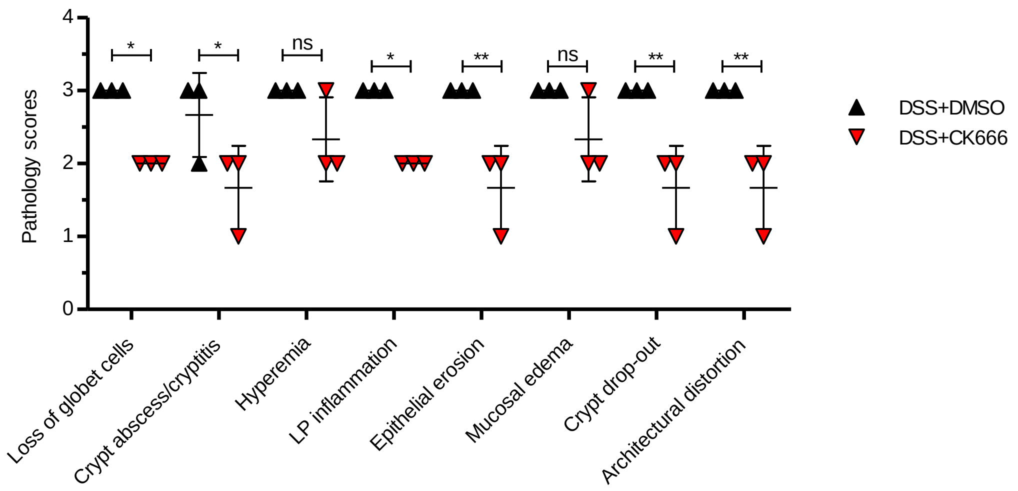

Figure S6. CK666 ameliorates mucosal damage in DSS-induced colitis in mice. Colons from colitic mice treated with vehicle (DMSO) or CK666 during the 7-day DSS colitis period were scored for loss of goblet cells, cryptitis, hyperemia, lamina propria (LP) inflammation, epithelial erosion, mucosal edema, crypt drop-out and architectural distortion (n=3; 2-way ANOVA with Bonferroni's correction). *p<0.05; **p<0.01. The scoring system was adapted from *Shukla et al., 2018, Scientific Reports*.
